# Methods for Preserving Cellular and Milk Fat Globules RNA from Human Milk Samples

**DOI:** 10.21203/rs.3.rs-6968867/v1

**Published:** 2025-07-01

**Authors:** Zhengfei Li, Nicole Fischbein, Flora Jin, Sarah Nyquist, Kimberly O’Brien, Nadav Ahituv, Valerie Flaherman, Yarden Golan

**Affiliations:** Cornell University; University of California; University of California; Gladstone Institutes; Cornell University; University of California; University of California; Cornell University

**Keywords:** Human milk, RNA preservation, RNAlater, milk fat globules, milk cells, RNA integrity, transcriptomics, biobanking

## Abstract

Human breast milk contains RNA in various fractions, including milk cells and milk fat globules (MFG), making it a valuable resource for studying lactation physiology. However, preserving RNA integrity, especially in low-resource or at-home collection settings, is challenging due to rapid RNA degradation.

This study aimed to evaluate and optimize RNA preservation methods for BM cells and MFGs, including a simplified protocol using RNAlater for stabilization prior to freezing. Human milk samples (n = 16) were collected from lactating participants and either frozen directly (standard practice) or mixed with RNAlater (1:1, v/v) before freezing. RNA was extracted from separated milk cell and MFG fractions and assessed for concentration, quality (RNA quality number-RQN and 28S/18S ratio), and gene expression (*ACTB, LALBA, PRLR, PTPRC*) using qPCR.

MFG fractions consistently yielded higher RNA concentrations than milk cells. Samples preserved with RNAlater showed significantly improved RNA quality, particularly in the MFG fraction, compared to those frozen without RNAlater. Gene expression was largely stable across preservation methods, though the immune marker gene *PTPRC* was reduced in RNAlater-treated samples, suggesting shifts in immune cell RNA content. Delays in RNAlater addition led to declining RQN values in milk cell fractions, underscoring the need for prompt stabilization.

These findings demonstrate that RNAlater pre-freezing stabilization enhances RNA quality and yield, especially from MFGs, and supports its use for lactocytes gene expression analysis. This approach provides a practical, scalable solution for RNA preservation in clinical and field research, including remote and low-resource settings.

## Introduction

RNA is present in all fractions of human milk, including milk cells, which contain cellular RNA; the aqueous phase, which contains cell-free RNA and RNA from exosomes; and milk fat globules (MFG), which harbor cytoplasmic crescents derived from the lactocyte cells that secrete them[1,2]. Over the years, various studies have employed different fractions of milk for RNA extraction, depending on their specific research objectives[3–6]. Among these, MFG RNA is the easiest to measure, as this fraction is particularly rich in RNA and provides direct access to the lactocyte transcriptome without requiring cell sorting [1,4,7,8].

In contrast, RNA extracted from milk cells represents a mixed population, predominantly lactocytes, alongside varying proportions of immune cells [9–12]. Because of this heterogeneity, bulk RNA extraction and sequencing from milk cells can make it challenging to determine the cellular origin of the observed gene expression signals. To address this limitation, some researchers perform cell sorting before analysis[11–15]. However, this approach can be technically demanding and, in some cases, the number of sorted cells may be insufficient for downstream analyses, depending on the sample quality. An alternative strategy involves sequencing the entire milk cell pellet and applying computational deconvolution methods to infer cell proportions in the original sample [6]. These deconvolution algorithms typically rely on reference datasets from single-cell RNA sequencing (scRNA-seq) to predict the cellular composition and disentangle transcriptional signals from complex mixtures. This approach can be particularly useful when sorting is not feasible, offering a computational avenue for exploring the transcriptomic contributions of different cell types in milk samples.

In recent years, human milk cells have been increasingly studied to gain insights into the mammary gland’s biology during lactation and to investigate the immune cells present in milk. Notably, these cells have been shown to remain viable and capable of growing in culture, further expanding their utility for research [10]. While some studies have utilized cells immediately following isolation from milk[9], it is also possible to cryopreserve these cells after isolation, allowing long-term storage and making longitudinal or large-scale studies more feasible [10]. In this paper, we present a detailed, step-by-step protocol for processing fresh milk samples, which enables the efficient storage of milk fat globules (MFG) for the extraction of high-quality RNA, as well as the isolation and cryopreservation of human milk cells.

However, in many study sites, especially in low- and middle-income countries (LMICs), or in large clinical studies involving at-home sample collection, the ability to utilize protocols requiring laboratory equipment and trained personnel is often limited. The same is true for most clinical settings, where such resources may not be readily available, limiting the ability to obtain high-quality RNA from milk samples for research. To address these challenges, we developed a simplified protocol to preserve human milk samples using RNAlater, a commercially available RNA stabilization reagent (ThermoFisher). This method provides a practical solution for preserving samples in settings with limited access to laboratory infrastructure, enabling researchers to securely store milk for subsequent downstream RNA extraction. Herein, we share results from our analysis demonstrating the efficiency of RNAlater in preserving RNA quality from both MFG and milk cells, highlighting its potential utility for studies conducted in resource-constrained environments.

## Methods

### Participant cohort

Milk samples for this study were collected at the UCSF Infant Growth and Milk Supply study. The institutional review board of the University of California, San Francisco, approved the study (#19–29297). Written informed consent was obtained from all study volunteers.

### Sample collection

Fresh human milk samples were self-collected by participants into sterile containers at home and brought to the lab on ice (n = 15), or were expressed in the lab lactation room (n = 1). Samples were refrigerated (4°C) at home until they were brought to the lab, or were held on ice. For each collection, mothers were instructed to use a new/autoclaved/sterile pump kit, wash their hands before pumping, and empty both breasts. Ten to thirty milliliters of milk were collected from each breast for analysis in the lab. The time of milk expression reported by the mother and the time of sample processing initiation reported by the lab member processing the sample were documented.

### Samples processing

Each sample received in this study was aliquoted into 5 or more DNA LoBind tubes (Eppendorf, Catalog # 022431021). In addition, 2 ml of milk was mixed with 2 ml RNAlater (ThermoFisher, Catalog # AM7021) and was aliquoted into two 2 ml DNA LoBind tubes. In this study, we tested 16 human milk samples; a portion of each sample was mixed with RNAlater (BMR), and a separate portion of each sample was frozen at the same time frame without mixing with RNAlater (BM). Once received and processed at the lab, samples were kept in −80°C until RNA extraction (samples stored for 256–652 days). Sample characteristics are listed in **Table S1**.

### RNA extraction

To better understand the effect of mixing samples with RNAlater on RNA measurement in the different milk fractions, we centrifuged each sample after thawing to separate the milk cells (Cells/C) from the milk fat globules (MFG/F) and extracted RNA from each fraction separately. A total of 64 RNA samples were extracted and tested in this study (from each milk sample, we extracted 4 RNA samples coming from cells and MFG with and without RNAlater). On the day of analysis, samples were thawed on ice and centrifuged for 10 min, 4°C at 15,000g. Upper layer of MFG (F) was separated into a new tube and was mixed with Qiazol lysis reagent (Qiagen, Catalog # 79306). Supernatant was carefully removed using a pipette, and the cell pellet (C) was examined, mixed with Qiazol lysis reagent and transferred to a new tube. Samples were vortexed to lyse the fat and cells, and RNA was extracted using the RNeasy Plus Universal Mini Kit (Qiagen, Catalog # 73404) following the manufacturer’s protocol, skipping the TissueLyzer step, which was determined to be unnecessary for these types of samples.

### RNA quality measurements

RNA concentration was measured using Nanodrop^™^ One Spectrophotometer (Invitrogen Catalog # 13-400-518). The 5200 Fragment Analyzer system was used to measure RNA Quality Number (RQN) and 28s/18s ratio of each RNA sample.

### qPCR for gene expression

Reverse transcription was performed with 500 ng of total RNA using qScript cDNA Synthesis Kit (Quantabio, 95047) following the manufacturer’s protocol. qRT-PCR was performed on QuantStudio 6 Real Time PCR system (ThermoFisher) using PerfeCTa^™^ SYBR^®^ Green FastMix^™^, Low ROX^™^ (QuantaBio, 95074) with the primers for detection of *ACTB, LALBA, PRLR* and *PTPRC* listed in **Table S2**. To evaluate if gene detection/expression is different between the treatments (BMR vs. BM), we used the ΔΔCt method to estimate fold change in RNA content with *ACTB* serving as the housekeeping gene, and the BMR samples with the shorter time until process (0.5h) as the control. Fold change = 2^−ΔΔCt^. ΔΔCt = ΔCt (RNA extracted from each sample) - ΔCt (RNA extracted from BMR sample processed after 0.5h from the same milk fraction). ΔCt (mRNA) = Ct (mRNA of each sample)-Ct (ACTB of the same sample).

### Statistical analysis:

Statistical analysis and figures generation were performed using Prism 10 for macOS (Version 10.4.1). Two-way ANOVA with Tukey’s multiple comparisons test was used to compare the fractions and treatments for each parameter tested (between-groups comparison). In addition, we performed a paired Student’s t-test analysis to look for differences in concentration and fold change expression between the treatments (BMR vs BM) in each fraction separately. Significance for all statistical analyses was set at p ≤ 0.05.

## Results

### Isolation of human milk cells for cryopreservation or downstream analysis

We present a detailed protocol designed to extract RNA from milk fat globules (MFG) while ensuring the preservation of MFG RNA in high quality for future analyses (**Appendix 1**). Unlike the protocol described by Lemay et al.[4], our method avoids implementing a hard spin step, which may compromise the viability of milk cells intended for cryopreservation or subsequent in vitro experiments. Additionally, to maintain the integrity of the fat globules and their original concentration within the sample, we deliberately refrained from introducing additional PBS before the first centrifugation step, as previously suggested [16,17].

For researchers seeking to eliminate potential signals from infiltrated, intact cells within the MFG fraction, we recommend adding supplementary washing steps of the fat layer with PBS after the initial centrifugation step [4]. Our protocol is intentionally streamlined and requires only a cold centrifuge and basic laboratory equipment, making it suitable for use in research settings equipped with the resources necessary for cell isolation and processing. Details outlining the required equipment, reagents, and step-by-step procedures are provided in **Appendix 1**.

#### RNA integrity of human milk declines sharply with freezing, but can be preserved by mixing milk with RNAlater before freezing

Over the decades, human milk samples collected in large cohorts have typically been stored at temperatures ranging from − 20°C to −80°C until analyzed[6,18]. However, this traditional freezing process often results in significant degradation of the RNA content in milk, rendering these samples unsuitable for sequencing. To address this issue, we conducted an experiment to assess the ability of RNAlater, an RNA-preserving reagent, to maintain RNA quality and integrity in milk when mixed in a 1:1 ratio before freezing.

**Figure 1:**
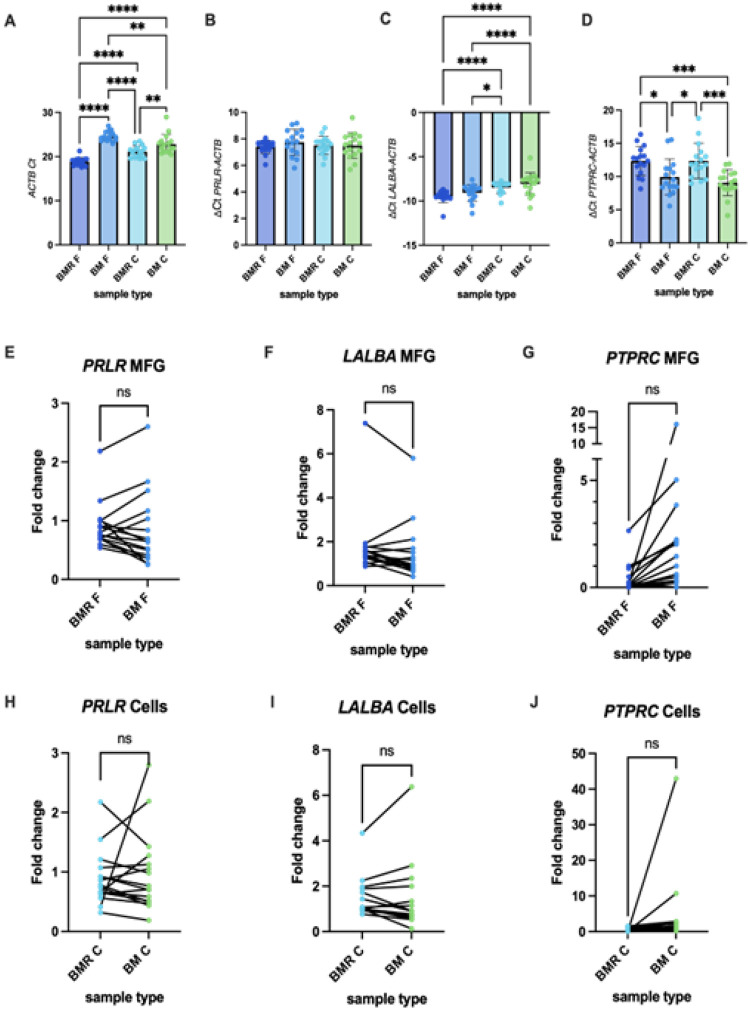
Comparison of RNA quality and yield in human milk samples (n=16) frozen with and without RNAlater. (A) RNA concentrations (ng/μL) measured from each fraction are shown on the Y-axis for samples extracted from MFG and milk cells under different conditions (as indicated on the X-axis). Asterisks represent significant differences between groups based on Tukey’s multiple comparison test. (B) Paired t-test analysis comparing RNA concentrations from MFG (F) samples preserved with RNAlater (BMR F) versus without RNAlater (BM F), extracted from the same milk sample. (C) Paired t-test analysis comparing RNA concentrations from milk cells (C) preserved with RNAlater (BMR C) versus without RNAlater (BM C), from the same milk sample. (D) RNA Quality Number (RQN) values and (E) 28S/18S ratios of samples from each fraction are displayed on the Y-axis for the conditions indicated on the X-axis. Asterisks represent significant differences between groups using Tukey’s multiple comparison test. (F and G) Scatter plots depicting how RQN values change when samples are mixed with RNAlater at increasing time points post-milk expression. The Y-axis represents RQN scores, while the X-axis shows the time elapsed (hours) before RNAlater addition. Simple linear regression analysis was performed, and the mean regression line is shown along with the 95% confidence interval (CI). Significant differences are indicated as *p < 0.05, **p < 0.01, ***p < 0.001, ****p < 0.0001.

RNA concentration was significantly higher in the MFG fraction compared to milk cells across all tested conditions (Fig. 1A). Notably, all samples started from either 1 mL of milk mixed with RNAlater or 1.5–2 mL of milk without RNAlater. When comparing RNA concentrations between the MFG samples preserved with RNAlater (BMR F) and those frozen without RNAlater (BM F), we observed higher RNA yields in the BMR F group (on average by 60%), despite starting with a smaller milk volume (Fig. 1B). No significant difference in concentration was observed between the cell treatments, with a mean difference of 5 ng/μl (SD = 26.3) (Fig. 1C). These findings suggest that the addition of RNAlater before freezing might enhance RNA yield in MFG. Notably, the 260/230 ratios observed in the NanoDrop reads were very low in samples treated with RNAlater and exhibiting low RNA concentrations (**Table S1**). Interestingly, milk cell pellets observed after freezing with RNAlater (BMR C) were visibly much larger than those from samples frozen without RNAlater (BM C) (**Figure S1**). Despite this increase in pellet volume, RNA yield did not increase, possibly due to the pelleting of additional non-cellular components, likely salts or other debris introduced by the high salt content of the RNAlater solution.

In addition, our results demonstrated that RNA extracted from both the MFG fraction (BM F) and milk cells (BM C) of frozen samples without RNAlater had substantially lower RNA Quality Number (RQN) scores and 28S/18S ratios compared to samples mixed with RNAlater before freezing (BMR F and BMR C, respectively) (Fig. 1D and 1E). RQN scores in all samples frozen without RNAlater were consistently below the recommended levels for RNA sequencing, highlighting significant RNA degradation, and the lower 28S/18S ratios observed in these samples further confirmed the extent of RNA degradation in the absence of RNAlater stabilization. These results indicate that pre-freezing stabilization with RNAlater significantly improves both RNA quality and integrity in milk samples.

We next aimed to determine the time frame after expression that samples should be mixed with RNAlater to provide data to inform sample collection instructions in a clinical research setting. In MFG, we observed that RQN values remained high when samples were mixed with RNAlater up to two hours after milk expression (Fig. 1F). After this time, some samples showed lower RQN values, although there was no significant correlation between the time elapsed since milk expression and the RQN, suggesting that samples mixed at later time points might still be of good quality for sequencing. In the milk cell fraction, however, a significant reduction in RQN was observed over time since collection, as indicated by a simple linear regression model (Fig. 1G). Despite this, the earliest samples (that were mixed with RNAlater two hours or less after expression) still exhibited relatively lower RQN scores compared to their matched MFG samples (Fig. 1G). This suggests that factors other than time since expression, such as RNase activity in an individual sample, might affect the RNA integrity.

##### Impact of RNAlater on gene expression patterns across milk fractions.

**Figure 2:**
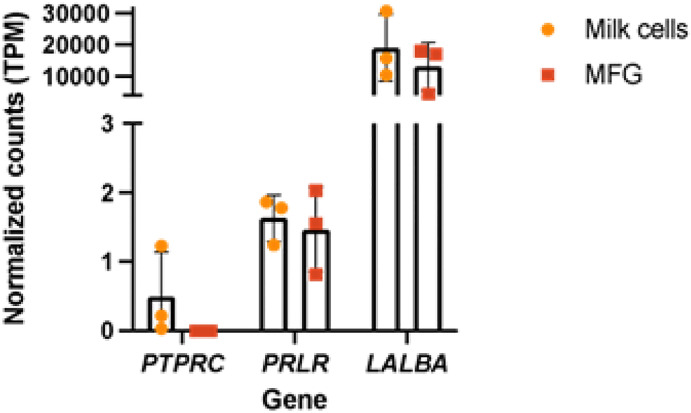
Gene expression profiles from RNA sequencing of milk fat globules (MFG) and milk cells from three fresh milk samples. Read counts were normalized to transcripts per kilobase million (TPM) and are plotted on the Y-axis. Gene names for transcripts (*PRLR, LALBA*, and *PTPRC*) are displayed on the X-axis. Bars represent mean TPM values, and error bars denote variability across the three samples.

For our qPCR analysis, we targeted three genes with different expression patterns based on our previous RNA-sequencing analysis comparing milk cells and MFG RNA [2]. The *ACTB* (beta-actin) was used as a housekeeping gene, *PRLR* (prolactin receptor), which is expressed in low levels in MFG and milk cells, *LALBA* (alpha-lactalbumin), which has very high expression in these fractions during lactation, and *PTPRC* (protein tyrosine phosphatase receptor type C, also known as CD45), which is not expressed in MFG and is expressed in milk cells samples with a high portion of immune cells (Fig. 2).

**Figure 3:**
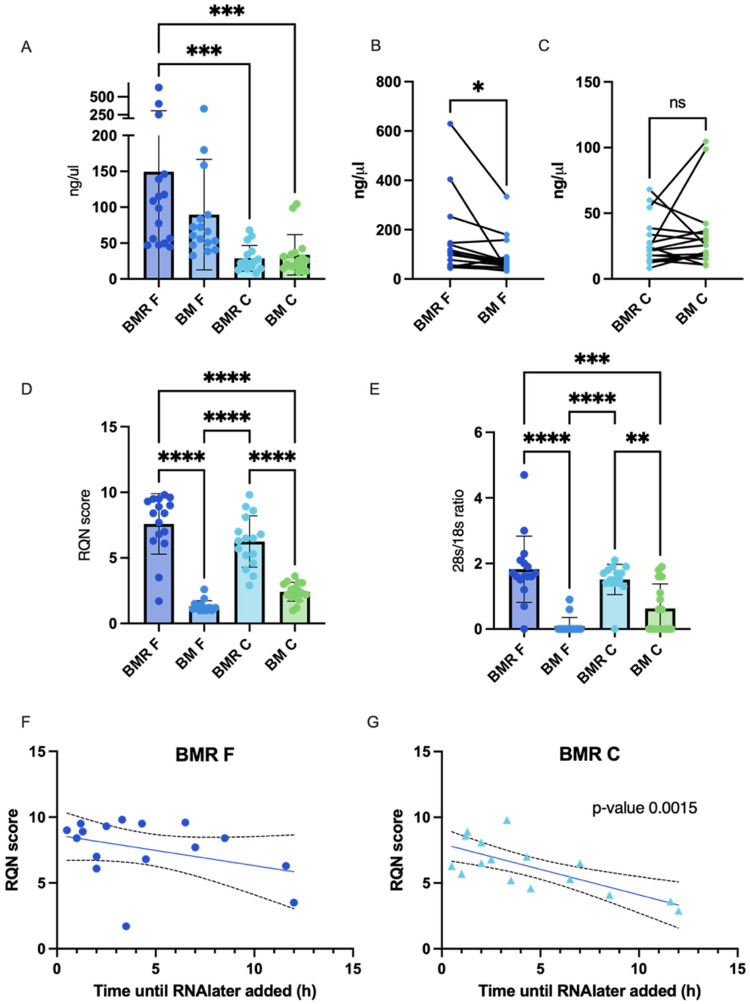
Effect of preservation method on gene expression measured by qPCR. (A) Ct values of the *ACTB* housekeeping gene across different milk fractions (MFG [F] and cell [C]) and preservation methods: BM (standard freezing method) and BMR (with RNAlater). (B–D) ΔCt values for *PRLR(B), LALBA* (C), and *PTPRC* (D), calculated as gene Ct – *ACTB* Ct. Lower Ct or ΔCt values indicate higher gene expression. Data are presented as mean ± SEM with individual sample points overlaid. (E-G) Fold change in expression of *PRLR* (E), *LALBA* (F), and *PTPRC* (G) genes in the MFG fraction. (H-J) Fold change in expression of *PRLR*(H), *LALBA* (I), and *PTPRC* (J) genes in the milk cells fraction. Fold change was calculated relative to expression in samples preserved in RNAlater within 0.5 hours post-expression (reference). Statistical comparisons reflect differences between BMR and BM treatments. Lines connect data from the same sample across different milk fractions. Each dot represents the mean value of 2–3 experimental measures from each sample. Significant differences are indicated as *p < 0.05, **p < 0.01, ***p < 0.001, ****p < 0.0001.

Using specific primers for these genes, we aimed to evaluate whether low RNA Quality Number (RQN) scores affect the expression patterns of these genes and whether mixing milk with RNAlater impacts their expression or proportions across different milk fractions. We observed significantly higher Ct values for the housekeeping gene *ACTB* in milk samples frozen without RNAlater (BM) compared to those mixed with RNAlater (BMR), suggesting that RNA degradation in the BM samples reduces the detectability of genes (Fig. 3A). To normalize for these differences, we adjusted the expression of target genes to *ACTB* by calculating ΔCt values. For *PRLR*, ΔCt values did not differ between preservation methods (BMR vs BM) or milk fractions (MFG vs milk cells) (Fig. 3B). This indicates no impact of RNAlater on this gene’s expression, which is considered very low (Ct values of 25–33). In contrast, ΔCt values for *LALBA* were lower (indicating higher expression) in the MFG fraction compared to milk cells, regardless of the preservation method, and no differences between processing methods (BMR vs BM) were observed within each fraction (Fig. 3C). For *PTPRC*, ΔCt values were higher in BMR compared to BM, reflecting reduced expression of this immune-related gene when RNAlater was used (Fig. 3D). Surprisingly, *PTPRC* expression was not higher in the milk cell fraction compared to the MFG fraction in either processing group, despite milk cells being known to contain more immune cells (Fig. 3D). Additionally, ΔCt values for *PTPRC* in BMR F were higher than in cells without RNAlater (BM C) but not higher than in cells preserved with RNAlater (BMR C), suggesting that RNAlater may alter the immune cell population or reduce their abundance during freezing. Fold change analysis, derived using the ΔΔCt method (comparing test samples to a reference sample mixed with RNAlater 0.5 hours post-collection), revealed no significant differences in expression of any gene (Fig. 3E–J). Furthermore, we found no significant correlation between the expression of *PRLR, LALBA*, or *PTPRC* and the time elapsed between milk expression and RNAlater mixing (BMR) or direct freezing (BM). These results suggest that RNAlater improves RNA quality and detectability while subtly impacting immune-related gene expression, possibly due to changes in immune cell populations during preservation. The reduction of PTPRC expression after RNAlater treatment should be investigated in relation to specific cell markers, particularly when the study focuses on specific immune cell populations.

## Discussion

In this study, we explored methods for preserving RNA quality in human milk samples, specifically focusing on separating the milk fat globules (MFG) and milk cell fractions, utilizing RNAlater as a stabilization reagent for this biospecimen. Our results underline the challenges posed by traditional milk freezing protocols, as samples frozen without RNAlater exhibited substantial RNA degradation, evident by RQN or RIN scores and 28S/18S ratios below recommended thresholds for RNA sequencing. This highlights the need for stabilization methods that preserve RNA integrity in milk samples, particularly in studies that seek to utilize transcriptomic analyses, but do not have the capacity to process fresh milk samples for RNA extraction.

The degradation of RNA upon freezing is often attributed to the inherent presence of RNases and lactoferrin in human milk [19–22]. The use of commercially available preservative reagents was previously shown to be effective in preserving bacterial DNA integrity in human milk samples [23]; however, to our knowledge, the use of preservatives for RNA stabilization in human milk has not been tested before. Without timely stabilization, freezing alone does not completely inhibit RNase activity, leading to rapid degradation of RNA, as shown by our results and others [6,24]. Previous studies have sequenced RNA from milk samples stored at −80°C or samples snap-frozen in liquid nitrogen, despite low RNA integrity scores, and found that milk protein transcripts were the most highly expressed [6,24], as expected in milk samples. However, using samples with low RNA can affect the expression profile of other genes, and the expression levels observed don’t necessarily reflect the expression at sample collection [25,26]. It was also previously suggested that cow’s milk has an inherent low RIN score of MFG RNA[24], but our results suggest that this might be a result of the storage methods used, and it is possible that preserving cow’s milk with RNAlater can also improve its RNA integrity.

We demonstrated that mixing milk with RNAlater before freezing significantly improves both RNA yield and quality in the MFG fraction, despite starting with smaller milk volumes. This suggests that RNAlater enhances RNA extraction efficiency from MFG. These results were in line with using RNAlater reagent for preserving RNA from adipose tissue[27]. However, for milk cells, while the pellet size increased following RNAlater stabilization, RNA yield did not improve. This observation raises the possibility that non-cellular components, such as salts introduced by RNAlater, contribute to pellet expansion. These findings emphasize the importance of considering reagent-associated artifacts when optimizing sample preservation protocols. In addition, further studies should explore if the addition of RNAlater changes the composition of the milk cell pellet.

Our analysis of gene expression patterns via qPCR revealed that RNA degradation in samples frozen without RNAlater reduced the detectability of housekeeping genes such as *ACTB*. These findings are in line with previous studies that found that expression patterns might change due to low RIN scores [26]. Normalized gene expression analysis showed that *PRLR* and *LALBA* expression remained unaffected by the preservation method (BMR vs BM), with *LALBA* exhibiting higher expression in the MFG fraction compared to milk cells. Interestingly, *PTPRC* expression was reduced in RNAlater-preserved samples (BMR C and BMR F), which may reflect changes in immune cell populations during sample freezing and preservation. Contrary to expectations, no higher *PTPRC* expression was observed in milk cells compared to MFG fractions in either processing group, raising questions about how immune-related RNA profiles might shift under different preservation methods. This effect warrants closer investigation, particularly in studies targeting immune cell-specific markers. It is notable that fold change analyses and correlations with time until RNAlater mixing did not yield significant differences for any of the tested genes across the 12 h time interval studied, indicating that immediate stabilization with RNAlater has no adverse effects on gene-specific transcriptional profiles but is crucial for maintaining RNA integrity overall.

Our time-lapse results highlight the importance of timely sample processing, suggesting a two-hour window for RNAlater addition as optimal for preserving RNA integrity in MFG samples, while also suggesting that factors beyond just time, such as RNase activity, sample handling, and others, may contribute to RNA degradation in the milk cell fraction.

In conclusion, stabilizing human milk samples with RNAlater prior to freezing is an effective approach for preserving RNA quality and enhancing RNA yield, particularly from MFG. Our work underscores the value of RNAlater as a tool for ensuring high-quality RNA recovery from milk fractions, particularly in studies where transcriptomic analyses are integral. Future investigations should examine how RNAlater interacts with specific cell populations and RNA profiles, particularly for immune-related markers, and other downstream assays such as single-cell RNA sequencing, to further refine preservation protocols and optimize their applicability across diverse research settings.

## Supplementary Files

This is a list of supplementary files associated with this preprint. Click to download.

- TableS1.xlsx
- Table2qpcrprimers.xlsx
- FigureS1.png
- Appendix1.docx
